# Overcoming Hypoxic-Resistance of Tumor Cells to TRAIL-Induced Apoptosis through Melatonin

**DOI:** 10.3390/ijms150711941

**Published:** 2014-07-04

**Authors:** You-Jin Lee, Ju-Hee Lee, Ji-Hong Moon, Sang-Youel Park

**Affiliations:** Biosafety Research Institute, College of Veterinary Medicine, Chonbuk National University, Jeonju, Jeonbuk 561-756, Korea; E-Mails: lee267@hanmail.net (Y.-J.L.); ishe1205@nate.com (J.-H.L.); carisma-j-h@hanmail.net (J.-H.M.)

**Keywords:** melatonin, TRAIL (tumor necrosis factor-related apoptosis-inducing ligand), HIF-1α (hypoxia inducible factor-1α), PHD2 (prolyl-hydroxylase 2), MTP (mitochondrial transmembrane potential), Bax translocation

## Abstract

A solid tumor is often exposed to hypoxic or anoxic conditions; thus, tumor cell responses to hypoxia are important for tumor progression as well as tumor therapy. Our previous studies indicated that tumor cells are resistant to tumor necrosis factor-related apoptosis-inducing ligand (TRAIL)-induced cell apoptosis under hypoxic conditions. Melatonin inhibits cell proliferation in many cancer types and induces apoptosis in some particular cancer types. Here, we examined the effects of melatonin on hypoxic resistant cells against TRAIL-induced apoptosis and the possible mechanisms of melatonin in the hypoxic response. Melatonin treatment increased TRAIL-induced A549 cell death under hypoxic conditions, although hypoxia inhibited TRAIL-mediated cell apoptosis. In a mechanistic study, hypoxia inducible factor-1α and prolyl-hydroxylase 2 proteins, which increase following exposure to hypoxia, were dose-dependently down-regulated by melatonin treatment. Melatonin also blocked the hypoxic responses that reduced pro-apoptotic proteins and increased anti-apoptotic proteins including Bcl-2 and Bcl-xL. Furthermore, melatonin treatment reduced TRAIL resistance by regulating the mitochondrial transmembrane potential and Bax translocation. Our results first demonstrated that melatonin treatment induces apoptosis in TRAIL-resistant hypoxic tumor cells by diminishing the anti-apoptotic signals mediated by hypoxia and also suggest that melatonin could be a tumor therapeutic tool by combining with other apoptotic ligands including TRAIL, particularly in solid tumor cells exposed to hypoxia.

## 1. Introduction

Melatonin and its metabolites [1] have multiple functions in biological rhythms, sleep induction, and even immunomodulation [2,3]. The biological effects of melatonin are signaled in both receptor-dependent [4] and receptor-independent manners [5,6]. Various levels of melatonin secretion coincide with sleep disorders, depression, stress, and some forms of cancer [7,8,9,10]. Novel specific roles of melatonin have been investigated, particularly in association with various types of cancers [7,11]. Factors that impact tumor growth change the levels of circulating melatonin; these include ischemia that leads to tissue hypoxia, which plays an important role in the ability of tumor cells to adapt, survive, and metastasize. According to a recent report, melatonin possesses anti-angiogenic activity by inhibiting stabilization of hypoxia inducible factor-1α (HIF-1α) in human tumor cell lines [12].

Hypoxia is a common tumor stressor. In particular, rapidly-growing solid tumor cancer cells are often hypoxic at their center. Hypoxia exhibits anti-apoptotic potential by dysregulating a variety of apoptosis signaling pathways [13] and decreases the effects of anticancer drugs, including TRAIL, in solid tumor cells [14]. HIF-1, a transcriptional factor composed of α- and β-subunits, is a key regulator of metabolic adaptation to hypoxia [15]. Targets of HIF-1α include cytokines and growth factors, angiogenesis-promoting genes, cell cycle progression, glucose uptake and metabolism, and cell survival. Thus, HIF-1α has been implicated as an oncogene that is overexpressed in human cancer cells. Appropriately, blocking HIF-1α as a therapeutic target alone or in combination with chemotherapeutic reagents has been explored [16,17]. Under normoxic conditions, HIF-1α is hydroxylated by prolyl-hydroxylases (PHDs) and binds with pVHL, followed by polyubiquitination, which is the main target of proteasomal degradation [18]. However, PHDs lose their activity under hypoxic conditions, so unhydroxylated HIF-1α is stabilized and further modified to obtain transactivational capacity. It is commonly accepted that reactive oxygen species, which are generated primarily by mitochondria due to an inappropriate metabolic phenotype under hypoxia, provoke the signaling pathways to inactivate PHDs [19,20,21].

TRAIL induces apoptosis by first binding to its membrane death receptors including death receptor 4 (DR-4) and DR-5. This causes the formation of a death-inducing signaling complex, which includes the receptors, the adaptor protein, FADD, and caspase-8. Although TRAIL is a member of the tumor necrosis factor (TNF) family, it has some notable differences compared with TNF-α and FasL. Unlike Fas, the TRAIL receptors DR-4 and DR-5 are widely expressed [22], which means that most tissues and cell types are potential TRAIL targets [22,23]. TRAIL induces apoptosis in a wide variety of tumor cells but not in most normal cells. Recent preclinical studies have demonstrated that repeated systemic administration of recombinant TRAIL protein effectively limits tumor growth without any serious side effects [24,25]. Therefore, considerable attention has been paid to TRAIL as a promising therapy for human cancers. However, many studies have indicated that tumor cells exposed to hypoxia are resistant to TRAIL-induced cancer cell apoptosis [14,26,27] and overcoming inhibition by hypoxia has been a target in solid tumor anticancer therapy using the TRAIL protein [28,29]. Melatonin possesses anti-angiogenic activity by inhibiting stabilization of HIF-1α in the HCT116 cell line [12,30].

Based on these results, we hypothesized that melatonin blocks hypoxic inhibition and enhances TRAIL activity in TRAIL-resistant tumor cells exposed to hypoxia. In the present study, we examined the inhibitory effect of melatonin and its possible mechanisms in hypoxia-mediated TRAIL resistant tumor cells and also suggested that melatonin may be a therapeutic strategy for anti-cancer therapy in combination with TRAIL, particularly in solid tumor cells exposed to hypoxia.

## 2. Results and Discussion

### 2.1. Melatonin Enhanced TRAIL (Tumor Necrosis Factor-Related Apoptosis-Inducing Ligand)-Induced Human Lung Cancer Cell Death under Hypoxic Conditions

**Figure 1:**
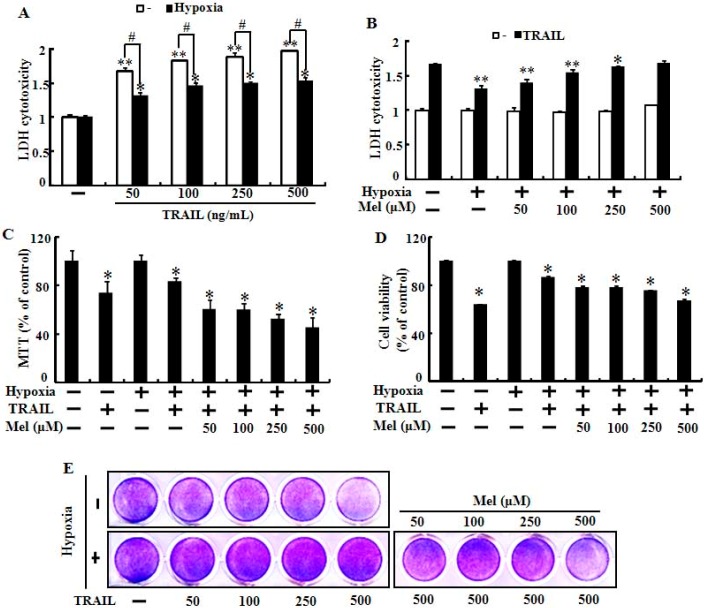
Melatonin increases TRAIL (tumor necrosis factor-related apoptosis-inducing ligand)-induced cell death in hypoxia. (**A**) Cells were treated with TRAIL in a dose-dependent manner under normoxic/hypoxic condition (1% of O_2_) for 24 h. Cytotoxicity was determined by lactate dehydrogenase (LDH) release; (**B**) LDH released from cells under normoxia/hypoxia, with or without melatonin treatment in a dose-dependent manner and exposed to TRAIL (500 ng/mL) for 24 h; (**C**) The cells were treated with or without melatonin in a dose-dependent manner and exposed to TRAIL for 24 h to determine cell viability under normoxia or hypoxia. Cell viability was measured by 3-(4,5-dimethylthiazol-2-yl)-2,5-diphenyltetrazolium bromide (MTT) assay; (**D**) Cell viability under the conditions described in (**C**) was determined by crystal violet staining; and (**E**) Crystal violet stained cells were photographed with a scanner. The bar graph shows mean ± standard error. ** *p* < 0.01, * *p* < 0.05, significant difference between control and each treatment group. ^#^ *p* < 0.01, significant difference between pretreated with normoxia and hypoxia. The data represent samples from 3 different experiments.

**Figure 2:**
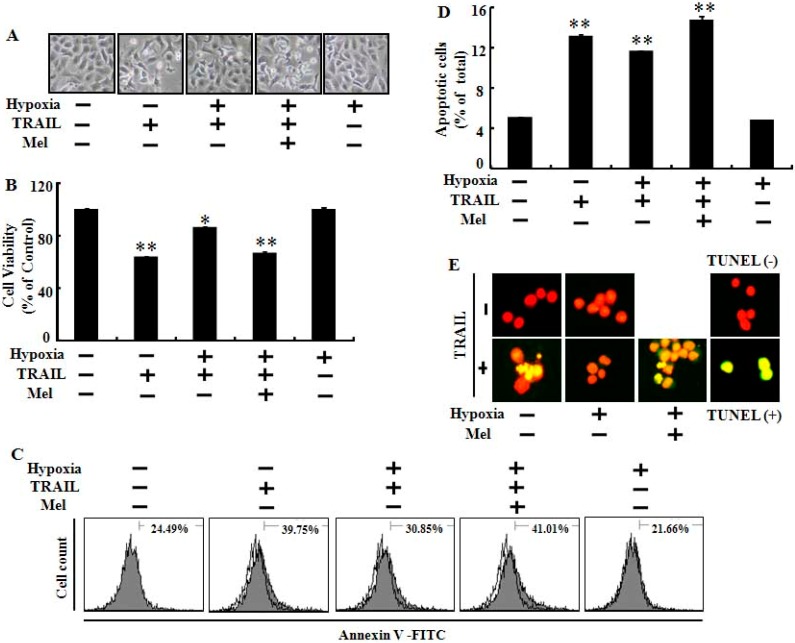
Melatonin enhances TRAIL-induced apoptosis against inhibition of hypoxia. A549 cells were treated with or without 500 μM melatonin and exposed to TRAIL (500 ng/mL) under normoxia/hypoxia for 24 h. (**A**) Cell morphology was photographed with a light microscope; (**B**) Cell viability was determined by crystal violet staining; (**C**) Annexin V-positive cells were detected by flow cytometry; (**D**) Apoptotic cells were measured by the Annexin V assay. Bar graph indicates the average of Annexin V-positive cells; and (**E**) Representative immunofluorescence images of terminal deoxynucleotidyl transferase dUTP nick and labeling-positive (green) cells. The cells were counterstained with propidium iodide (red) to show all cell nuclei. Bar graph indicates mean ± standard error. ** *p* < 0.01, * *p* < 0.05, significant difference between control and each treatment group. The data represent samples from 3 different experiments.

Hypoxia is a well-characterized component of the lung carcinoma microenvironment and inhibits cell apoptosis mediated by anti-cancer agents including TRAIL. To examine the effects of melatonin on hypoxic-inhibition of TRAIL-induced cancer cell apoptosis, we pretreated with melatonin and co-cultured lung cancer cells with TRAIL exposed to hypoxia or normoxia (Figure 1). The LDH (lactate dehydrogenase) cytotoxicity assay showed that TRAIL activity was inhibited by exposure to hypoxia and that the melatonin treatment gradually recovered the reduced LDH cytotoxicity in tumor cells exposed to hypoxia (Figure 1A,B). Treatment with 500 µM melatonin completely attenuated the hypoxic inhibition of TRAIL-induced A549 cell apoptosis (Figure 1B). The MTT (3-(4,5-dimethylthiazol-2-yl)-2,5-diphenyltetrazolium bromide) and cell viability assays also demonstrated that melatonin mitigated the inhibition of TRAIL activity mediated by hypoxia (Figure 1C–E). We also evaluated the effects of melatonin and/or TRAIL in human lung cancer cells exposed to hypoxia via various cell death and viability assay techniques including Terminal uridine deoxynucleotidyl transferase dUTP nick end labeling (TUNEL) assay and Annexin V flow cytometry. These results indicated that melatonin treatment sensitized lung cancer cells against hypoxic inhibition by TRAIL-induced apoptosis (Figure 2). Microscopic photographs and the crystal violet assay results showed that hypoxia inhibited TRAIL-induced apoptosis and that pretreatment with melatonin abolished the inhibitory effects of hypoxia in TRAIL-mediated tumor cell death (Figure 2A,B). The Annexin V assay also demonstrated that TRAIL-mediated apoptotic cells were inhibited by exposure to hypoxia and that inhibition was blocked by melatonin (Figure 2C,D). These results were confirmed by measuring Alexa Fluor 488-labeled anti-BrdUrd with the TUNEL assay microscopic images (Figure 2E). Taken together, these results indicate that melatonin significantly increased TRAIL-induced tumor cell death under hypoxia and suggest that melatonin treatment may regulate the hypoxia signal pathway that protects lung cancer cells against TRAIL-induced cell death.

### 2.2. Melatonin Down-Regulates Hypoxia-Related Protein Expression

**Figure 3:**
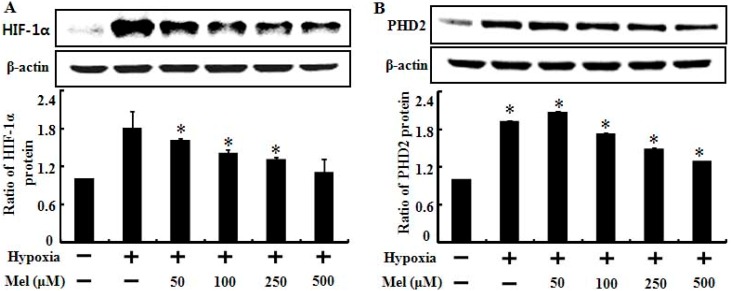
Melatonin down-regulates hypoxia-induced HIF-1α (hypoxia inducible factor-1α) and PHD2 (prolyl-hydroxylase 2) protein expression. Effect of melatonin treatment on cells under hypoxia (1% of O_2_) on protein levels. Total protein was isolated, and protein expression was examined by Western blot analysis. (**A**) A549 cells were treated with melatonin under normoxia/hypoxia. The treated cells were assessed for HIF-1α expression and densitometric values are shown below the Western blots. Results were normalized to β-actin; (**B**) PHD2 expression levels were analyzed and shown by the bar graph. The bar graph indicates the mean ± standard error. * *p* < 0.05, significant difference between control and each treatment group. The data represent samples from 3 different experiments.

According to recent reports, melatonin suppresses tumor angiogenesis by inhibiting the stability of HIF-1α under hypoxic conditions [12,30,31]. In the present study, we examined the expression pattern of HIF-1α and HIF PHD2, an enzyme that promotes degradation of HIF-1α in human lung cancer cells, treated with melatonin and hypoxia (Figure 3). As shown in Figure 3A, HIF-1α protein levels increased significantly following exposure to hypoxia and the high HIF-1α protein level was dose-dependently decreased by melatonin treatment. This result was supported by a previous report stating that melatonin inhibits HIF-1α stabilization under hypoxic conditions [30]. We also tested whether the reduction of HIF-1α was mediated by PHD2 regulation by melatonin, and the result showed that hypoxia treatment highly induced PHD2 expression and that the PHD2 protein was decreased slightly but dose-dependently by melatonin (Figure 3B). These results indicate that melatonin-mediated reduction of HIF-1α is not correlated with HIF-1α degradation induced by HIF-1α hydroxylation via PHD2 but may be regulated by melatonin itself.

### 2.3. Melatonin Up-Regulates Pro-Apoptotic Signals Mediated by Hypoxia

**Figure 4:**
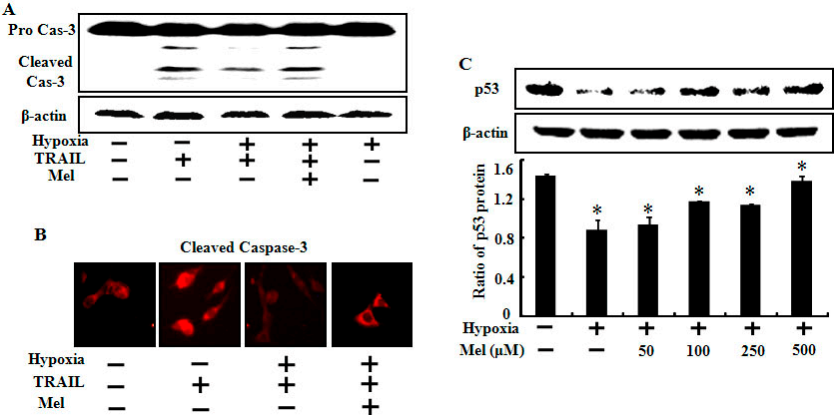
Effects of melatonin on expression of various death signals involved in TRAIL-induced apoptosis. A549 cells were treated with or without 500 μM melatonin and exposed to TRAIL (500 ng/mL) under normoxia/hypoxia for 24 h. (**A**) Treated cells were assessed for caspase-3 cleavage by Western blot analysis. β-actin was used as the loading control; (**B**) Treated cells were immunostained with cleaved caspase-3 antibody (red); and (**C**) A549 cells were treated with melatonin in a dose-dependent manner under hypoxia (1% of O_2_). Western blot analysis of p53 expression levels in A549 cells treated as described. Densitometric values of the p53 protein are shown. Bar graph indicates the mean ± standard error. * *p* < 0.05, significant difference between control and each treatment group. The data represent samples from 3 different experiments.

To understand the molecular mechanism of enhanced TRAIL sensitivity to hypoxic tumor cells by melatonin, we examined caspase-8 and p53 expression levels in A549 lung cancer cells treated with TRAIL, melatonin, and hypoxia. The results showed that cleaved caspase-8 increased by TRAIL treatment and that increased active caspase-8 was markedly blocked under hypoxic conditions (Figure 4A). The inhibition of active caspase-8 mediated by hypoxia was completely restored by melatonin treatment (Figure 4A). The treated cells were immunostained with cleaved caspase-3 antibody, and the results indicated that melatonin attenuated the hypoxic inhibition of TRAIL-induced apoptosis (Figure 4B). The Western blot assay also showed that the p53 protein expression level decreased following hypoxia, and that melatonin dose-dependently increased the lowered p53 protein level (Figure 4C). Our results are supported by a recent study reporting that melatonin inhibits cell proliferation and prevents DNA damage in both normal and transformed cells by activating p53 [32].

### 2.4. Melatonin Down-Regulates Anti-Apoptotic Signals Mediated by Hypoxia

**Figure 5:**
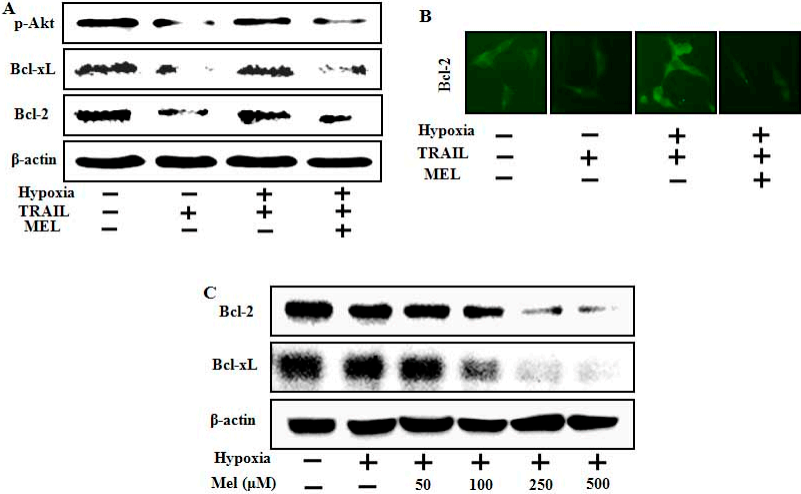
Effects of melatonin on expression of various survival factors involved in hypoxia. (**A**) A549 cells were pretreated with melatonin and hypoxia, and then exposed to 500 ng/mL TRAIL. Phospho-Akt, Bcl-xL and Bcl-2 expression levels were determined by Western blot analysis; (**B**) Representative images of Bcl-2 expression; and (**C**) A549 cells were treated with melatonin in a dose-dependent manner under hypoxic conditions (1% of O_2_). Western blot analysis of the Bcl-2 protein and β-actin was used as the loading control.

Many tumor cells acquire resistance via exposure to hypoxia, which up-regulates anti-apoptotic proteins and survival signals [33]. To study the effect of melatonin on hypoxia-mediated anti-apoptotic proteins and survival signals, we examined Akt activation and Bcl-xL and Bcl-2 protein expression using Western blot analysis and immunofluorescence staining. The results showed that TRAIL inhibited Akt activation, inhibition was blocked by hypoxia exposure, and melatonin recovered the Akt inactivation level following TRAIL treatment. The Bcl-xL and Bcl-2 proteins were also down-regulated by melatonin treatment in hypoxic cells, which highly expressed these proteins (Figure 5A). We also confirmed that melatonin inhibited Akt activation mediated by hypoxia through immunofluorescence staining (Figure 5B). We evaluated the expression pattern of anti-apoptotic proteins in hypoxic tumor cells treated with melatonin alone, and the data showed that melatonin dose-dependently decreased Bcl-xL and Bcl-2 protein expression (Figure 5C). These data indicate that melatonin can overcome the hypoxic TRAIL-resistant tumor cells by down-regulating anti-apoptotic proteins including Bcl-xL and Bcl-2.

### 2.5. Melatonin Inhibited Hypoxia-Mediated Regulation of Mitochondrial Dysfunction and Bax Translocation

**Figure 6:**
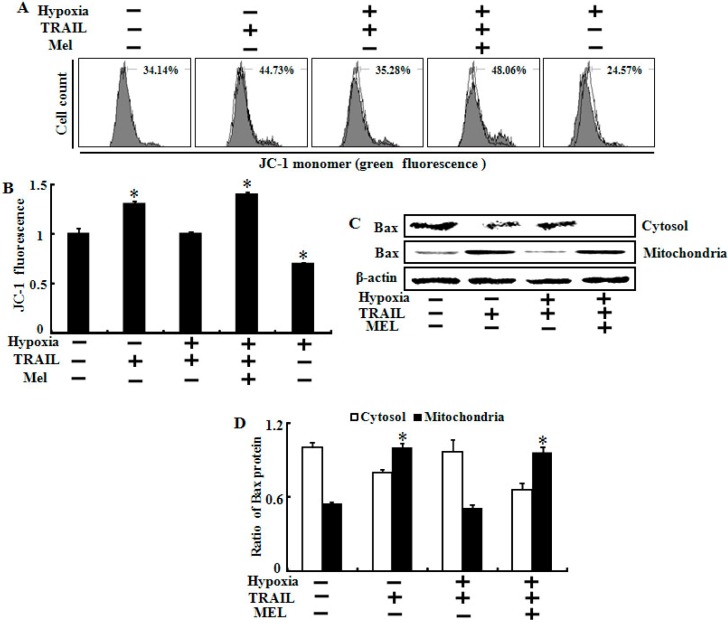
Melatonin inhibited hypoxia-mediated regulation of mitochondrial dysfunction and Bax translocation. (**A**) A549 cells were treated with or without 500 μM melatonin and exposed to TRAIL (500 ng/mL) under normoxia/hypoxia for 24 h. Treated cells were counted using JC-1 mono (green) by flow cytometry. Mark represents the population of JC-1 monomeric cells; (**B**) Bar graph indicates the green fluorescence intensity of the JC-1 mono form; (**C**) Treated cells were homogenized in mitochondrial buffer, and then the isolated cytosolic and mitochondrial extracts were analyzed by Western blotting using antibody against the Bax protein; and (**D**) Densitometric values of the Bax protein are shown. β-actin was used as the loading control. Bar graph indicates the mean ± standard error. * *p* < 0.05, significant difference between control and each treatment group. The data represent samples from 3 different experiments.

Many studies have indicated that the inhibitory effect of hypoxia on TRAIL-induced apoptosis may be involved in preventing Bax translocation and protecting the MTP [26,34]. We investigated the effect of melatonin on hypoxia-mediated protection of mitochondrial dysfunction including the MTP and Bax translocation using JC-1 fluorescence and Western blot assays (Figure 6). Hypoxia prevented the TRAIL-mediated increase in green fluorescent cells, indicating a lowered MTP and mitochondrial dysfunction, and co-treatment of melatonin converted cells to the same condition as the TRAIL alone treatment, which showed a lowered MTP under hypoxic conditions (Figure 6A,B). As shown in Figure 6C, melatonin blocked the hypoxic inhibition of Bax translocation in the cytosol to mitochondria induced by TRAIL, and showed the same results in the densitometer analysis of the Western results (Figure 6D). These results demonstrate that melatonin blocks hypoxic inhibition of TRAIL-induced apoptosis by regulating MTP and Bax translocation.

### 2.6. Discussion

Rapidly growing tumor cells exposed to hypoxia are resistant to many anticancer therapies including chemo and radiotherapy. Many researchers have investigated ways to overcome this resistance for successful anticancer therapy. In our previous studies, hypoxia inhibited TRAIL-induced tumor cell death in lung and colon cancer cells. We hypothesized that melatonin treatment would block the hypoxic inhibition and enhance TRAIL activity in TRAIL-resistant tumor cells exposed to hypoxia. In the present study, melatonin recovered TRAIL activity from TRAIL-resistant hypoxic cells and enhanced TRAIL-induced apoptosis under hypoxic conditions (Figure 1 and Figure 2). Melatonin inhibited the stability of HIF-1α in time- and concentration-dependent manners [12,30,35]. We hypothesized that melatonin would suppress the PHD2 and HIF-1α proteins and the data showed that the PHD2 and HIF-1α proteins were down-regulated by melatonin (Figure 3). HIF-1α activity under normoxic conditions is modulated by oxygen-dependent hydroxylation of proline residues by PHD. PHDs and HIF form a self-regulating loop so that decreased pO_2_ would be expected to increase HIF-1α activity, which, in turn, increases PHD2 expression [36]. A previous report led us to believe that melatonin-mediated HIF-1α and PHD2 down-regulation is the possible mechanism by which melatonin enhanced TRAIL-induced apoptosis under hypoxic conditions.

Although HIF-1α down-regulation is the main pathway for enhancing this mechanism, target genes within TRAIL signaling and hypoxia are poorly understood. In the present study, we investigated several key target genes and clarified their underlying mechanisms (Figure 4, Figure 5 and Figure 6). First, our data indicate that melatonin increased p53 and active caspase-3 protein expression under hypoxic conditions (Figure 4). Activation of the p53 tumor-suppressor pathway is a critical mediator of melatonin and its anticancer effects [32]. Melatonin recovered the p53 protein expression level reduced by hypoxia in a concentration-dependent manner in our study. Second, we found that Bcl-xL and Bcl-2 played a pivotal role restoring TRAIL activity during hypoxia. Although the Bcl-xL and Bcl-2 proteins were highly expressed in control cells, hypoxia maintained the level of both proteins, whereas melatonin down-regulated their expression levels dose-dependently. Many studies have revealed that high levels of Bcl-xL and Bcl-2 may or may not inhibit TRAIL-induced apoptosis under hypoxia and propose that a decrease in anti-apoptotic proteins could be a possible mechanism for our present results [37,38,39,40]. Finally, we suggest that the matrix metalloproteinase (MMP) and bax translocation are a recovery mechanism for melatonin against hypoxic inhibition. Many studies have indicated that the change in the MMP and Bax translocation caused by various stimuli enhances tumor cell apoptosis [38,41,42]. Our data revealed that melatonin may regulate mitochondrial dysfunction and Bax translocation to restore the hypoxic resistant responses against TRAIL-induced apoptosis. Further studies should be done using animal models to test whether melatonin can suppress the hypoxic inhibition of TRAIL-induced apoptosis in solid tumor cells.

## 3. Experimental Section

### 3.1. Cell Culture and Reagents

The A549 human lung adenocarcinoma cell line was obtained from the American Type Culture collection (Rockville, MD, USA). The cells were cultured in RPMI-1640 (Gibco BRL, Grand Island, NY, USA) medium containing 10% fetal bovine serum (Gibco) and antibiotics (0.1 mg/mL penicillin-streptomycin). The cells were incubated in an atmosphere containing 5% CO_2_ at 37 °C in 12-well plates. The cells were pretreated with or without melatonin and exposed to normoxic or hypoxic conditions for the indicated times and further treated with soluble recombinant human TRAIL protein (200 ng/mL) for 18 h.

### 3.2. Hypoxic Conditions

A sealed chamber was used to culture the lung cancer cells at low oxygen tension (1%). A gas mixture of 1% O_2_, 5% CO_2_ and 94% N_2_ was added to the sealed chamber, and ambient air was evacuated through an outlet tube. Oxygen flow was allowed to stream through the chamber for 2–3 min to maintain the desired oxygen tension inside the chamber. The culture plates were incubated in sealed chambers containing 1% O_2_ at 37 °C. The cells were incubated at 37 °C in a 95% humidified atmosphere with 5% CO_2_ under normoxic conditions (21% O_2_ tension). Two controls (normoxia and hypoxia) were used in this experiment. The hypoxia control was handled in the same type of sealed unit as used for 1% O_2_.

### 3.3. Lactate Dehydrogenase (LDH) Assay

Cytotoxicity was assessed by the LDH assay in supernatant medium using a LDH Cytotoxicity Detection kit (Takara Bio, Inc., Tokyo, Japan), according to the manufacturer’s protocol. LDH activity was determined by measuring absorbance at 490 nm using a microplate reader (Spectra Max M2, Molecular Devices, Sunnyvale, CA, USA).

### 3.4. Crystal Violet Assay

Cell viability was evaluated by crystal violet staining. Briefly, cells were exposed to a staining solution (0.5% crystal violet in 30% ethanol and 3% formaldehyde) for 10 min at room temperature and washed four times with water. The stained cells were lysed with 1% sodium dodecyl sulfate (SDS), and absorbance was measured at 550 nm. Cell viability was calculated based on the relative dye intensity compared with that of the control.

### 3.5. MTT (3-(4,5-Dimethylthiazol-2-yl)-2,5-diphenyltetrazolium bromide) Assay

The colorimetric 3-[4,5-dimethylthiazol-2-yl]-2,5-diphenyl tetrazolium bromide (MTT) assay was performed to quantify the effect of different test agents on cell viability and to standardize gel loading. Briefly, 2 × 10^4^ cells/well were seeded in a 96-well microplate at a final volume of 200 μL, incubated overnight, and treated with test agents for 24 h. A 10 μL aliquot of 5 mg/mL MTT (Sigma, St. Louis, MO, USA) was added to the cells 3 h prior to the completion of the treatment, and the cells were incubated for an additional 3 h. The MTT solution was removed and replaced with 100 μL dimethyl sulfoxide, and the plates were agitated for 3 min. Optical density was determined at a wavelength of 570 nm.

### 3.6. Annexin V Assay

Apoptosis was assessed by the Annexin V assay in detached cells using the Αnnexin V Assay kit (Santa Cruz Biotechnology, Santa Cruz, CA, USA), according to the manufacturer’s protocol. Annexin V measurements were obtained by measuring fluorescence at an excitation wavelength of 488 nm and an emission wavelength of 525/530 nm using a Guava EasyCyte HT instrument (Millipore, Milford, MA, USA).

### 3.7. Terminal Uridine Deoxynucleotidyl Transferase dUTP Nick End Labeling (TUNEL) Assay

TUNEL analysis was performed to measure the degree of cellular apoptosis using an *in situ* ApoBrdU DNA Fragmentation Assay kit (BioVision, Mountain View, CA, USA) following the manufacturer’s instructions. Cells were fixed by suspension in 70% (*v*/*v*) ethanol and stored at −20 °C overnight. The samples were then incubated with DNA-labeling solution (10 μL reaction buffer, 0.75 μL TdT enzyme, 8 μL BrdUTP, 31.25 μL of dH_2_O) for 1 h at 25 °C. Each sample was then exposed to an antibody solution consisting of 5 μL Alexa Fluor^®^ 488-labeled anti-BrdU antibody with 95 μL of rinse solution and allowed to react for 20 min. Images were captured at ×20 objective using a fluorescence microscope (Nikon Eclipse 80i; Nikon Corp., Tokyo, Japan).

### 3.8. Western Blot Analyses

For Western blot analysis, A549 cells were lysed in lysis buffer (25 mM *N*-2-hydroxyethylpiperazine-*N*-2-ethane sulfonic acid (HEPES); pH 7.4, 100 mM NaCl, 1 mM ethylenediaminetetraacetic acid (EDTA), 5 mM MgCl_2_, 0.1 mM Dithiothreitol (DTT), and protease inhibitor mixture). Equal amounts of protein were separated by SDS-polyacrylamide gel electrophoresis for 1.5 h at 100 V, and the membranes were blotted by wet transfer (150 V, 90 min, 4 °C) onto nitrocellulose membranes. The membranes were then blocked with 5% dry fat milk in phosphate buffered saline containing 0.05% Tween 20 (Bio-Rad, Hercules, CA, USA) (PBST) for 1 h at room temperature. The membranes were probed with a primary antibody for 1 h at room temperature in PBST containing 1% nonfat dry milk. Primary antibodies used for immunoblotting were cleaved caspase-3 (Cell Signaling Technology, Danvers, MA, USA), HIF-1α (BD Biosciences, San Diego, CA, USA), PHD2 (Abcam, Cambridge, MA, USA), p-Akt (Epitomics, Burlingame, CA, USA), p53, Bcl-2, Bcl-xL, and Bax (Santa Cruz Biotechnology), DR-5, and β-actin (Sigma). After washing with PBST, bound primary antibody was detected with horseradish peroxidase-conjugated secondary antibodies (Bio-Rad), and the blots were developed using an enhanced chemiluminescence detection system (GE Healthcare, Chalfont St Giles, UK). Images were examined using anFusion-FX7 imaging system (Vilber Lourmat, Marne-la-Vallée, France).

### 3.9. Cellular Fractionation

A549 cells were resuspended in mitochondrial buffer (210 mm sucrose, 70 mm mannitol, 1 mm EDTA, 10 mm HEPES), broken with a 26-gauge needle, and centrifuged at 700× *g* for 10 min. The post-nuclear supernatant was centrifuged at 10,000× *g* for 30 min. The pellet was used as the mitochondrial fraction, and the supernatant was used as the cytosolic fraction. Total protein was obtained and subjected to Western blotting.

### 3.10. Immunofluorescent Staining

Cells cultured on glass slides were fixed with cold acetone, blocked with 5% fetal bovine serum in Tris buffer solution and Tween 20 (TBST), and incubated with rabbit active caspase-3 antibody (Cell Signaling Technology) and mouse Bcl-2 antibody (Santa Cruz Biotechnology) overnight at 4 °C. After washing with TBST, the cells were incubated with goat anti-rabbit IgG conjugated with Alexa Fluor^®^ 546 (red) and goat anti-mouse IgG conjugated with Alexa Fluor^®^ 488 (green). Cells were washed with TBST, mounted with fluorescence mounting medium (Dako, Carpentaria, CA, USA), and observed under a fluorescence microscope (Nikon ECLIPSE 80i, Nikon Corp.). Images were acquired and processed using a Nikon digital camera and (Diagnostic Instruments, Sterling Heights, MI, USA) and Image J software (National Institute of Healthy, Bethesda, MD, USA).

### 3.11. Mitochondrial Transmembrane Potential (MTP) Assay

The change in the MTP was evaluated using the cationic fluorescent indicator JC-1 (Molecular Probes, Eugene, OR, USA), which aggregates in intact mitochondria (red fluorescence) indicating high or normal MTP and low MTP when it remains in monomeric form in the cytoplasm (green fluorescence). A549 cells were incubated in RPMI-1640 containing 10 μM JC-1 at 37 °C for 15 min, washed with phosphate buffered saline (PBS) and then transferred to a clear 96-well plate. JC-1 aggregate fluorescent emissions were measured at 583 nm at an excitation wavelength of 526 nm, and JC-1 monomer fluorescence intensity was measured at excitation and emission wavelengths of 525 and 530 nm, respectively, using a SpectraMax M2 (Molecular Devices) or Guava easyCyte HT System (Millipore).

### 3.12. Statistical Evaluation

All data are expressed as mean ± standard deviation, and the data were compared using Student’s *t*-test. A *p*-value < 0.05 was considered significant.

## 4. Conclusions

Our results show the blocking effect of melatonin in hypoxia-mediated TRAIL resistance of tumor cells and its possible mechanisms. The results suggest that melatonin may be a therapeutic strategy for anti-cancer therapy in combination with TRAIL, particularly in solid tumor cells exposed to hypoxia.
